# Uterine Disease

**Published:** 1881-05

**Authors:** Edward C. Mann


### UTERINE DISEASES.

BY EDWARD C. MANN, M. D.

It is now perhaps two years since in your journal I read an account of Viburnum prunifolium being employed by some Boston physician in uterine diseases. As many cases of diseases of women occurring in connection with nervous diseases are annually treated here, I desire to call attention to my own investigations with this comparatively new medicine. It appears to me to act directly and specifically upon the special nerves of the uterus as a true nerve sedative. I have had several very violent cases of congestive and neuralgic forms of dysmennorrhœa, in one case the dysmennorrhœa being accompanied by epileptiform convulsions of a very severe type, and in each and every case I have seen almost magical relief following the use of the fluid extract of Viburnum prunifolium. The case referred to, which was so severe that the intensity of the pain had worn out the unhappy sufferer and induced the epileptiform attacks, was completely cured in a few weeks by the combined use of the Viburnum prunifolium and the use of the constant current of electricity, the positive pole being applied to the hypogastric region, and the negative pole, to which was attached a cup-shaped electrode, directly to the uterus. The galvanic current has a powerful influence in suspending contractions of the uterus, and is also very efficacious when used locally over the ovaries, in controlling ovarian neuralgia. Previous to my using the Viburnum prunifolium I had been accustomed to rely on valerianate of zinc and fluid extract of gelsemium with the constant current of electricity, but since my first experience with the former drug I have used nothing else. Although I have not had occasion to use it in cases of threatened abortion, I should deem it worthy of use from its action on the ganglionic nerves of the uterus. I have failed to perceive any action on the general system, the whole force of the medicine appearing to be directed to the uterus and its system of nerves. When the pulse has been high from nervous excitement, and the temperature centers in the brain, have been temporarily paralyzed, allowing sudden rise in temperature, from nervous excitement, both pulse and temperature have fallen to the normal as the uterine pain has been relieved. It must be remembered, also, that my cases have been aggravated ones, many of my cases having been sent to Sunnyside on the verge of insanity. My conclusions, therefore are, that in Viburnum prunifolium we have a uterine sedative more powerful than any other in controlling dysmenorrhœa and uterine contractions, and that it probably acts by passing from the blood to the nerve centers, and is special in its effect upon the ganglionic nerve of the uterus.—*Boston Med. and Surg. Jour.*

